# Effects of Nutrition Education on Levels of Nutritional Awareness of Pregnant Women in Western Iran

**DOI:** 10.5812/ijem.9122

**Published:** 2013-07-01

**Authors:** Farnoush Fallah, Ahmad Pourabbas, Ali Delpisheh, Yousef Veisani, Mahdi Shadnoush

**Affiliations:** 1 Nutrition Research Center, School of Nutrition, Tabriz University of Medical Sciences, Tabriz, Iran; 2 Medical Education Research Center, Tabriz University of Medical Sciences, Tabriz, IR Iran; 3 Department of Clinical Epidemiology & Prevention of Psychosocial Injuries, Research Centre, Ilam University of Medical Sciences, Ilam, IR Iran; 4 Institute of Nutritional Researches, Shahid Beheshti University of Medical Sciences, Tehran, IR Iran

**Keywords:** Pregnancy, Awareness, Health Education

## Abstract

**Background:**

Maternal nutritional health, before and during pregnancy, influences the health status of herself and her developing fetus. Pregnancy is an important condition for improving nutritional knowledge.

**Objectives:**

The present study aimed at determining effects of nutrition education on levels of nutritional awareness of a representative group of pregnant women in Western Iran.

**Patients and Methods:**

A quasi-experimental intervention was undertaken on a random sample of pregnant women (n = 100) attending urban health centers in Ilam city (western Iran) during the year 2011 for prenatal care. A nutritional education program containing two to four lessons was undertaken for small groups of between six to ten women. Nutritional knowledge was assessed before intervention (pretest) and followed by two posttests within three weeks interval.

**Results:**

The awareness level of pregnant women about healthy nutrition was significantly increased from 3% before intervention to 31% after the nutritional education intervention (P < 0.001). This significant difference was independent from maternal characteristics of age and levels of literacy and in obese mothers in particular.

**Conclusions:**

A nutritional education intervention will have a positive effect on nutritional awareness of pregnant women.

## 1. Background

A healthy and balanced diet is quit important in life time and during pregnancy in particular. The maternal diet must provide sufficient energy and nutrients to meet the mother's usual requirements, as well as the needs of the growing fetus and enabling mother to maintain her own stores of nutrients required for fetal and infant health as well as for future breastfeeding practices. The main recommendation is to follow a healthy, balanced diet (1). Pregnancy is an occasion when women become more aware of the importance of healthy nutrition and seek for more nutrition-related information. Compared to the period before preconception and pregnancy, pregnant women are more eager to know what they should eat and what not (2). A poor pregnancy diet can lead to various nutritional deficiencies. Proper nutrition is a part of pregnancy that cannot be forsaken. A balanced diet full of whole grains, fruits and vegetables will help keep health throughout pregnancy (3). Inadequate nutrition, especially early in the pregnancy, may impair fetal brain development and cause abnormalities in endocrine functioning, organ development and the energy metabolism of child (4). Education is an important factor in health promotion. Determination of training needs is essential to achieve this goal (5). Knowledge is not behavior, but it can be a determining factor of dietary behavior (6). Various reports indicate that in most undeveloped countries recommended amounts of nutrients based on recommended daily allowance (RDA) by the mothers cannot be adequately received (7). Knowledge, attitudes and false beliefs are the main barriers of change behavior (8). Community health to a large extent depends on fetal growth in pregnancy and maternal health (9). Maternal weight gain program was merged into the Safe Motherhood Programs for the first time in Iran in March 2005 and subsequently began in September 2006 in eleven provinces of the country (10). According to previous studies nutrients intakes including folate, B6, A, D vitamins, iron, phosphorus, calcium, magnesium and zinc were found to be insufficient in Iranian pregnant women (11-13). In addition, no research has been found that surveyed effects of nutrition education on the Iranian pregnant women.

## 2. Objectives 

The aim of this study was to evaluate the effect of an education intervention on the awareness of healthy nutrition in a group of Iranian pregnant women.

## 3. Patients and Methods

Pregnant women in the first trimester of pregnancy, aged between 16 to 40 years who attended urban health centers for prenatal care in Ilam city (western Iran) in 2011 were randomly recruited. Sample size was calculated using n = z2p (1- p) d2 (P = 0.5, z = 1.96, β = 0.98) formula. To meet this sample size, 196 names and addresses were randomly appointed. Of them, 49 individuals were excluded from the study based on hematology results and family doctor advises. Meanwhile, 47 refused to take part and 100 agreed to participate by informed consent. The questionnaire was designed specifically for this study. It was developed by multidisciplinary research specialists including nutrition, health practitioner and midwifery. The questionnaire was structured and the answer options were predetermined by the research team. The questionnaire was deemed to have face validity and content validity by a team of experts in nutrition and health practitioner. The alpha Cronbach's test was used for the internal reliability of the questionnaire. The questionnaire included socio demographic characteristics of age and occupation, nutrition education topics of fruits and vegetables, whole grains, and low fat milks, blood groups, results of urinary and blood tests, vital sign measurements and history of diseases including diabetes, tuberculosis and cardiovascular. Meanwhile, there were some questions regarding healthy diet during pregnancy according to nutrition package. The study intervention was based on a cognitive model of health education to improve participant's nutritional knowledge and safe behaviors to follow a healthy balanced diet. The idea was to make pregnant women to get informed about what they should eat and what should avoid during pregnancy by a face-to-face education. The educational content was based on a nutrition package provided by the Iranian Ministry of Health and Medical Education. Initially, the participants' knowledge of healthy nutrition during pregnancy and their demographic characteristics were assessed by a baseline interview completing a validated questionnaire. Interventions of nutritional education containing two to four lessons were conducted either individually or in small groups of six to ten participants. A total of hundred participants attended at least two nutrition lessons during the interventions. Participants who attended fewer lessons were not included in the data analysis. Nutritional knowledge was assessed before intervention (pretest) and followed by two posttests within three weeks interval. Process of completion of questionnaires and giving education was performed by trained midwives throughout face to face interviews. Analyzing questioners before and after intervention, scores of three and one were awarded for healthy and unhealthy changes respectively. If no change was observed or the respondent was unsure, a score of two was given. Obtained scores were then summed to produce an overall score for dietary change. The maximum and minimum scores were 90 and 30 respectively. Scores between 30-50, 51-70 and 71-90 were considered as weak, moderate and good knowledge respectively. Means, standard deviations, and frequencies were computed. Mean scores of pre and post tests were calculated by independent sample t tests. All probability values of 0.05 or less were considered statistically significant.

## 4. Results

The average age of participants was 26.5 ± 5.6 years. Majority of them had an educational attainment of high school diploma or lower (88%). Almost all mothers (93%) were housewives and 78% had a marriage age of over 18 years (Table 1). In terms of nutritional status using body mass index (BMI), 41% had normal weight, 28% were overweight, 26% were obese and 5% were underweight. Only one woman had an abnormal blood pressure, one had diabetes, one had nephropathy, and six women had a history of cardiovascular disease. Urine analysis was normal for all women. In hematology analysis, 81% had a normal fasting glucose, 65% had a normal Hematocrit and 31% had a normal hemoglobin level. History of abortion was reported by 13 women. In terms of contraception, 56% did not use any contraceptive method, 23% consumed contraceptive pills and 21% used condom (Table 2). Before educational intervention, awareness levels were estimated to be weak (31%), moderate (66%) and good (3%). The corresponding rates after educational intervention were weak (6%), moderate (63%) and good (31%). The awareness level of pregnant women about healthy nutrition was significantly increased from 3% before intervention to 31% after the nutritional education intervention independent of their age or literacy levels (P < 0.01), (Tables 3 and 4).

**Table 1.** Characteristics of Women Participatingin Nutrition Education Program

	No. (%)
**Age group, y**	
< 20	10(10)
21-25	30(30)
26-35	50(50)
> 36	10(10)
**Educational levels**	
Primary School	50(50)
Secondary School	38(38)
Diploma	4(4)
University	8(8)
**Job status**	
Housewife	93(93)
Employee	7(7)
**Marriage age, y**	
< 18	22(22)
> 18	88(88)

**Table 2.** Frequency of Abnormal Indexes in Hematology Tests and Anthropometric Measurements

Hematology	No. (%)
**Fasting Blood Sugar (FBS)**	4 (4)
**Hematocrit (HCT)**	4 (4)
**Hemoglobin (HB)**	61(61)
**Body mass index (BMI)**	
Under weight	5(5)
Normal weight	41(41)
Over weight	28(28)
Obese	26(26)

**Table 3.** Assessment of Nutritional Knowledge Before and After Education Intervention

Levels of Knowledge	Before, No. (%)	After, No. (%)	P value
**Week^a^**	31(31)	6(6)	0.001
**Moderate^b^**	63(63)	63(63)	0.91
**Good^c^**	3(3)	31(31)	0.01

^a^Scores between 30-50

^b^Scores between 51-70

^c^Scores between 71-90

**Table 4.** Maternal Characteristics by Levelsof Nutritional Knowledge

Characteristics	Levels of Nutritional Knowledge			
	Week	Moderate	Good	P value
**Age, y, Mean ± SD**	29.39 ± 5.89	26.79 ± 5.55	28.33 ± 1.15	0.04
**Household's monthly income, (USD), Mean ± SD**	250 ± 119.72	287 ± 147.81	366±115.47	0.02
**Age marriage, y, Mean ± SD**	23.12 ± 6.07	22.98 ± 4.68	23.33 ± 4.50	0.41

## 5. Discussion

**Figure 1 .**
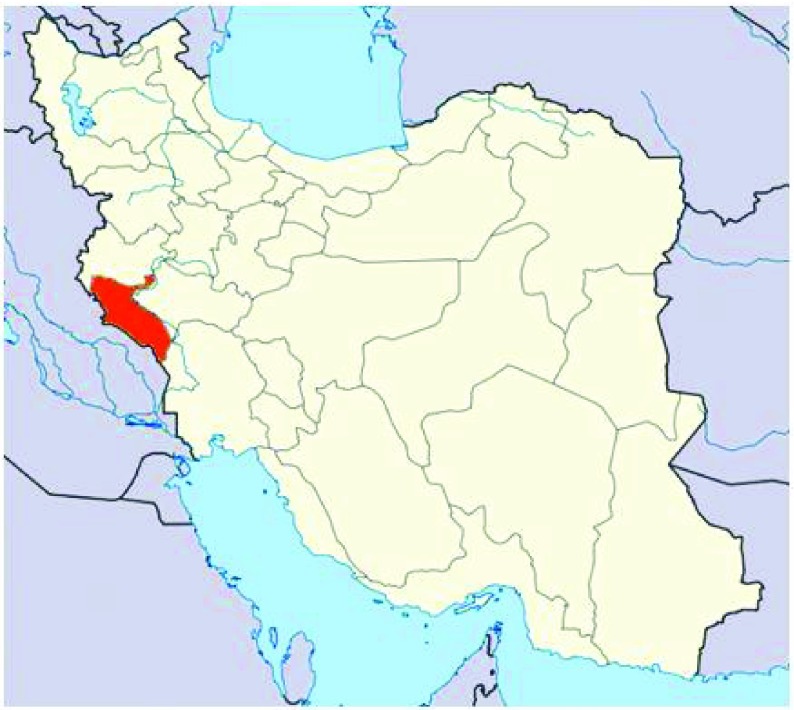
Map of Study Setting

Pregnancy is an important circumstance for rethinking and changing of nutritional habits. The present study was undertaken in the border city of Ilam, western Iran where is closed to Iraq country. Almost all participant mothers in the present study had been born during the eight years’ war between the two countries starting from 1981 onward. Meanwhile, nature of pregnancy, low household socio-economic status, poor incomes and unemployment as well as dusty polluted whether in this area make pregnant women more sensitive than other groups (Figure 1 ).

 Basically, the purpose of health education is to eliminate undesirable behaviors and replace them by appropriate and productive behavior leading to healthy life (9). This study sought to answer the question of whether nutrition education can influence a positive change to improve levels of nutritional knowledge of pregnant women. The present study showed significant improvements in awareness level of pregnant women who received at least two educational sessions on healthy nutrition in which it was significantly increased from 3% before intervention to 31% after the nutritional education intervention (P < 0.001). These results are similar to those found by Verbeke (2007) who found that education on nutrition and food consumption can resolve safety issues in population (14, 15). The present study also showed that the nutritional knowledge of pregnant women before intervention was very weak except for employed women. This result is in agreement with a recent reported survey (14). Another important finding was that in obese women, the weak, moderate and well educational knowledge levels after training were significantly increased by 19.3%, 7.7 % and 29.9 % respectively. A possible explanation for this might be that obese women compared with other women have given more attention to training. Poboeilk and colleagues have mentioned that pregnant women are more susceptible for obesity despite lower consumption of energy and other nutrients than standard during pregnancy period (16). Nutritional education programs during pregnancy especially for low-income pregnant, breastfeeding, and non-breastfeeding postpartum women are helpful steps to reduce material and spiritual costs in family and community. Nutrition education also is an appropriate intervention for food insecurity ( 3 ). The present study was limited by selecting participants from urban areas and few numbers of study settings. Pregnant women living in rural areas have more needs for nutritional education interventions. Despite these limitations, the study offers insight implication that nutrition education intervention can have a positive effect on nutritional awareness of pregnant women independent of their age or literacy and in obese women in particular.
